# Enzyme inhibition of dopamine metabolism alters 6-[^18^F]FDOPA uptake in orthotopic pancreatic adenocarcinoma

**DOI:** 10.1186/2191-219X-3-18

**Published:** 2013-03-14

**Authors:** Johanna Tuomela, Sarita Forsback, Laura Haavisto, Tero Vahlberg, Tove J Grönroos, Olof Solin, Merja Haaparanta-Solin

**Affiliations:** 1 MediCity/PET Preclinical Imaging, Turku PET Centre, University of Turku, Turku, 20520, Finland; 2 Pharmatest Services Ltd, Turku, 20520, Finland; 3 Radiopharmaceutical Chemistry Laboratory, Turku PET Centre, University of Turku, Turku, 20500, Finland; 4 Department of Biostatistics, University of Turku, Turku, 20520, Finland; 5 Accelerator Laboratory, Turku PET Centre, Åbo Akademi University, Turku, 20500, Finland

**Keywords:** Pancreas, Adenocarcinoma, [^18^F]FDOPA, AADC, COMT

## Abstract

**Background:**

An unknown location hampers removal of pancreatic tumours. We studied the effects of enzyme inhibitors on the uptake of 6-[^18^F]fluoro-l-3,4-dihydroxyphenylalanine ([^18^F]FDOPA) in the pancreas, aiming at improved imaging of pancreatic adenocarcinoma.

**Methods:**

Mice bearing orthotopic BxPC3 pancreatic adenocarcinoma were injected with 2-deoxy-2-[^18^F]fluoro-d-glucose ([^18^F]FDG) and scanned with positron emission tomography/computed tomography (PET/CT). For [^18^F]FDOPA studies, tumour-bearing mice and sham-operated controls were pretreated with enzyme inhibitors of aromatic amino acid decarboxylase (AADC), catechol-*O*-methyl transferase (COMT), monoamine oxidase A (MAO-A) or a combination of COMT and MAO-A. Mice were injected with [^18^F]FDOPA and scanned with PET/CT. The absolute [^18^F]FDOPA uptake was determined from selected tissues using a gamma counter. The intratumoural biodistribution of [^18^F]FDOPA was recorded by autoradiography. The main [^18^F]FDOPA metabolites present in the pancreata were determined with radio-high-performance liquid chromatography.

**Results:**

[^18^F]FDG uptake was high in pancreatic tumours, while [^18^F]FDOPA uptake was highest in the healthy pancreas and significantly lower in tumours. [^18^F]FDOPA uptake in the pancreas was lowest with vehicle pretreatment and highest with pretreatment with the inhibitor of AADC. When mice received COMT + MAO-A inhibitors, the uptake was high in the healthy pancreas but low in the tumour-bearing pancreas.

**Conclusions:**

Combined use of [^18^F]FDG and [^18^F]FDOPA is suitable for imaging pancreatic tumours. Unequal pancreatic uptake after the employed enzyme inhibitors is due to the blockade of metabolism and therefore increased availability of [^18^F]FDOPA metabolites, in which uptake differs from that of [^18^F]FDOPA. Pretreatment with COMT + MAO-A inhibitors improved the differentiation of pancreas from the surrounding tissue and healthy pancreas from tumour. Similar advantage was not achieved using AADC enzyme inhibitor, carbidopa.

## Background

Due to late diagnosis and lack of effective treatment, pancreatic adenocarcinoma has the worst prognosis of all of the gastrointestinal cancers [1]. Surgery is a possible curative approach if the cancer is detected early, but the exact location of the tumour is often difficult to determine. Current anatomy-based imaging procedures detect only indirect signs of invasive tumour growth such as pancreatic mass or ductal abnormalities. Therefore, there is a need for better functional imaging tools for the detection and localisation of pancreatic cancer.

The most frequently used positron emission tomography (PET) tracer for tumour imaging is the glucose analogue, 2-deoxy-2-[^18^F]fluoro-d-glucose ([^18^F]FDG). [^18^F]FDG is taken up into cells by glucose transporters, where it subsequently undergoes phosphorylation by hexokinase-1 into [^18^F]FDG-6-phosphate. This tracer is efficiently taken up by a variety of tumour cells and reflects increased glucose metabolism [2,3]. For pancreatic cancer, [^18^F]FDG has been useful in the evaluation of indeterminate pancreatic masses, staging of pancreatic carcinoma, detection of metastatic disease and differentiation of viable tumours from post-therapeutic processes like necrosis or scar tissue [4,5]. However, the diagnostic value of [^18^F]FDG in pancreatic cancer is limited since inflammatory processes such as pancreatitis and abscesses take up [^18^F]FDG as well. Chronic pancreatitis is recognised as the most common reason for false-positive [^18^F]FDG-PET tumour findings in the pancreas [2,6].

Investigation of the functional activity of the dopaminergic system is increasingly used in the evaluation of tumours of islet cell origin [7-12]. 6-[^18^F]Fluoro-l-3,4-dihydroxyphenylalanine ([^18^F]FDOPA), a fluorinated analogue of l-DOPA, an intermediate product in dopamine synthesis, is the most commonly used PET tracer in studies of hyperinsulinaemia and neuroendocrine tumours [7,8]. At present, the imaging of the pancreas using [^18^F]FDOPA is focused on the neuroendocrine nature of pancreatic cells [13]; however, the exocrine pancreas also contains dopamine [14]. According to previous immunohistochemical studies, exocrine cells can take up amine precursors such as l-DOPA, transport them across the cell membrane, convert them into dopamine by aromatic l-amino-acid decarboxylase (AADC) and store them in vesicles [15-17].

**Figure 1:**
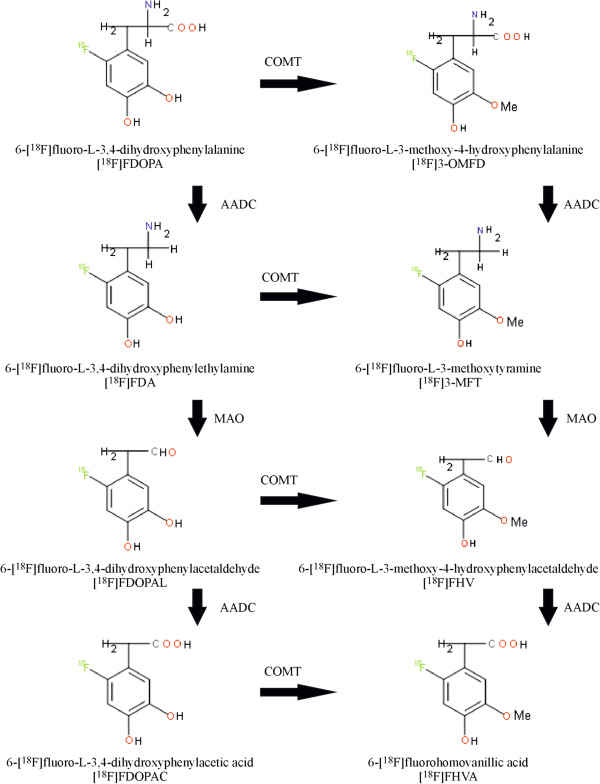
**Main metabolic pathways of [**^**18**^**F]FDOPA (modified from **[25]**).**

In the periphery, [^18^F]FDOPA is extensively metabolised to 6-[^18^F]fluoro-l-3-methoxy-4-hydroxyphenylalanine ([^18^F]3-OMFD) by catechol-*O*-methyltransferase (COMT) and by AADC to 6-[^18^F]fluoro-l-3,4-dihydroxyphenylethylamine (fluorodopamine, [^18^F]FDA), which is rapidly sulphated by phenolsulfotransferase to fluorodopamine sulphate [18]. [^18^F]FDA is also transformed by monoamine oxidase (MAO). The two isoforms of MAO, MAO-A and MAO-B, deaminate dopamine to produce 6-[^18^F]fluoro-l-3,4-dihydroxyphenylacetic acid ([^18^F]FDOPAC) before it is converted into 6-[^18^F]fluorohomovanillic acid ([^18^F]FHVA) by COMT [18,19]. [^18^F]FDA is also converted into 6-[^18^F]fluoro-l-3-methoxytyramine by COMT before being oxidised to [^18^F]FHVA by MAO (Figure 1) [20]. Carbidopa is a potent inhibitor of AADC. Based on the metabolism of FDOPA, carbidopa and COMT inhibitor are routinely used in a clinic prior to [^18^F]FDOPA injection into Parkinson's disease patients in order to minimise peripheral metabolism and to increase [^18^F]FDOPA concentrations in the brain [21]. Carbidopa also improves imaging of neuroendocrine tumours of the pancreas [22,23]. However, recent studies have shown that the use of enzyme inhibitors for other cancers of the pancreas, such as islet cell tumours, β cell hyperplasias and insulinomas, may hamper pancreatic uptake of [^18^F]FDOPA in addition to hindering its uptake by tumour cells [24].

The aim of this study was to improve the detection of pancreatic adenocarcinoma using PET. We used [^18^F]FDG and [^18^F]FDOPA and investigated the effects of enzyme inhibitors on [^18^F]FDOPA uptake, distribution and metabolism by means of PET/computed tomography (CT), autoradiography and chromatography in an orthotopic xenograft mouse model of pancreatic adenocarcinoma.

## Methods

### Cell culture

Human ductal pancreatic adenocarcinoma cells (BxPC3 cells) were cultured in RPMI-1640 medium supplemented with 10% heat-inactivated foetal calf serum and 2 mM of l-glutamine (all from Sigma-Aldrich Chemicals, Steinheim, Germany). The cells were maintained at 37°C in a humid atmosphere with 5% CO_2_. For orthotopic inoculation, the cells were trypsinised and suspended in Matrigel (BD Biosciences, San Jose, CA, USA) at a concentration of 10^6^ cells/mL and stored on ice. The viability of the cells was confirmed by trypan blue staining before and after inoculation.

### Orthotopic tumours

Five-week-old male immunodeficient nude mice (Athymic nude/nu Foxn1 mice, Harlan, The Netherlands) were used in this study. Animals were treated with analgesics (Temgesic® 0.1 mg/kg, mouse body weight 23.2 ± 1.9 g) and anaesthetised by isoflurane inhalation (3%, 200 mL/min). After laparotomy, 30 μL of cell suspension (3 × 10^4^ cells) was inoculated into the pancreatic body of the mice (*n* = 26). Mice were weighed twice per week, and their welfare was evaluated daily. Sham-operated nude mice (*n* = 22), which were inoculated only with Matrigel, were used as controls. Animals were sacrificed 35 or 42 days after cancer cell inoculation. All animal studies were approved by the Finnish Animal Ethics Committee. The institutional policy on animal experimentation fully meets the requirements defined in the National Institutes of Health’s *Guide for the Care and Use of Laboratory Animals* (NIH Publication no. 85–23, revised 1985).

### Preparation of radiopharmaceuticals

[^18^F]FDG was synthesised from mannosyl triflate using a nucleophilic method [26]. [^18^F]FDOPA was synthesised by electrophilic fluorination from the stannylated precursor 4,5-di-(1,1-dimethylethoxycarbonyl)oxy-N-formyl-2-trimethylstannyl-l-phenylalanine ethyl ester using high specific-radioactivity [^18^F]F_2_ as the labelling reagent [27,28]. The specific radioactivity of [^18^F]FDOPA was 5.8 GBq/μmol. Radiochemical purity exceeded 98% in every production batch [29].

### Pretreatments for [^18^F]FDOPA

In order to minimise the peripheral metabolism of [^18^F]FDOPA, animals were pretreated with enzyme inhibitors 10 min before injection of [^18^F]FDOPA. Sham-operated and tumour-bearing animals were divided into groups according to weight. Inhibitors and study groups are described in Table 1. Mice were treated with carbidopa (0.5 mg 0.25% carbomethyl cellulose sodium salt, Sigma-Aldrich Chemicals; *n* = 6 + 6, sham-operated and BxPC3), Ro-41-0960 (0.9 mg/150 μL in ethanol; *n* = 2 + 5), clorgyline (0.3 mg 0.25% carbomethyl cellulose sodium salt; *n* = 2 + 5) or a combination of Ro-41-0960 and clorgyline (*n* = 6 + 5). All enzyme inhibitors were purchased from Sigma-Aldrich Chemicals. Animals treated with 0.9% NaCl were used as controls (*n* = 6 + 5). Mice were either sacrificed for *ex vivo* analysis 10 min after injection of [^18^F]FDOPA or imaged using small animal PET/CT (Inveon Multimodality, Siemens Medical Solutions, TN, USA).

**Table 1:** ***Ex vivo *distribution of [**^**18**^**F]FDOPA in sham-operated and tumour-bearing mice (% ID/g)**

**Organ**	**Pretreatment**									
	**Vehicle**		**Carbidopa**		**Ro-41-0960**		**Clorgyline**		**Ro-41-0960 + clorgyline**	
	**None**^**a**^		**AADC**^**a**^		**COMT**^**a**^		**MAO-A**^**a**^		**COMT + MAO-A**^**a**^	
	**Sham**	**Tumour**	**Sham**	**Tumour**	**Sham**	**Tumour**	**Sham**	**Tumour**	**Sham**	**Tumour**
	***n*** = **6**	***n*** **= 5**	***n*** **= 6**	***n*** **= 6**	***n*** **= 2**	***n*** **= 5**	***n*** **= 2**	***n*** **= 5**	***n*** **= 6**	***n*** **= 5**
Pancreas^b^	7.3 ± 1.9	4.4 ± 2.6	24.9 ± 4.4	18.9 ± 11.9	6.8 ± 2.2	8,9 ± 2,7	7.1 ± 2.3	9.0 ± 2.3	20.4 ± 5.6	5.9 ± 4.7
Muscle	0.8 ± 0.2	1.1 ± 0.5	2.0 ± 0.5	2.2 ± 1.3	1.2 ± 0.1	1.6 ± 0.5	1.0 ± 0.0	0.9 ± 0.1	0.9 ± 0.2	1.0 ± 0.3
Liver	7.4 ± 0.6	9.7 ± 1.5	4.2 ± 0.7	4.6 ± 0.9	7.5 ± 0.5	8.2 ± 2.7	8.8 ± 0.6	7.6 ± 1.5	7.7 ± 0.8	7.6 ± 1.4
Blood	1.3 ± 0.3	1.5 ± 0.3	3.1 ± 0.5	3.3 ± 0.8	1.8 ± 0.2	2.7 ± 0.5	1.4 ± 0.0	1.3 ± 0.1	2.1 ± 0.4	2.5 ± 1.3
Heart	1.6 ± 0.4	1.9 ± 0.3	4.2 ± 0.9	4.3 ± 1.1	2.3 ± 0.3	3.5 ± 0.9	1.7 ± 0.1	1.6 ± 0.2	3.4 ± 0.2	3.6 ± 0.9
Kidneys	6.7 ± 1.6	7.8 ± 1.4	22.8 ± 3.9	19.2 ± 6.4	9.0 ± 3.1	22.0 ± 17.5	8.8 ± 4.8	6.3 ± 0.4	28.2 ± 10.7	25.9 ± 5.7
Spleen	1.6 ± 0.4	1.7 ± 0.3	4.4 ± 0.9	4.9 ± 1.5	2.8 ± 0.3	2.9 ± 0.8	1.8 ± 0.0	1.7 ± 0.3	2.9 ± 0.6	2.8 ± 0.9
Small intestine	6.2 ± 1.2	5.8 ± 1.0	4.0 ± 0.9	4.3 ± 0.8	7.0 ± 0.3	5.1 ± 0.8	10.1 ± 1.2	10.0 ± 1.9	11.1 ± 2.2	11.6 ± 1.3
Large intestine	1.2 ± 0.4	1.3 ± 0.4	1.8 ± 0.2	2.3 ± 0.5	1.5 ± 0.1	1.7 ± 0.2	2.1 ± 0.4	1.9 ± 0.3	2.9 ± 0.4	3.6 ± 1.6

Values are shown as mean ± standard deviation. ^a^Target enzyme; ^b^pancreata of BxPC3 tumour-bearing animals were measured with tumours.

### Small-animal PET/CT and image analysis

Six weeks after orthotopic inoculation of the BxPC3 cells, the uptake of [^18^F]FDG into pancreatic tumours (*n* = 14) was evaluated using small animal PET/CT. [^18^F]FDG (dose 9.0 ± 2.3 MBq) was injected into the tail veins of the animals under isoflurane anaesthesia. CT was used for attenuation correction of the PET data and anatomical reference following a 20-min static scan, which was acquired in list mode at 60 min after injection. The next day, selected mice (*n* = 4) were subjected to CT and a 20-min dynamic PET scan using [^18^F]FDOPA tracer. The injected dose was 6.8 ± 0.1 MBq, and the injected mass was 16 ± 10 ng/g, as calculated from the known specific radioactivity at the time of injection. Images were reconstructed using a 2D filtered backprojection with a 0.5-mm ramp filter. Data were collected for 20 min after injection of [^18^F]FDOPA, and the corresponding time-activity curves were calculated. Images were analysed using the Inveon Research Workplace software (v. 3.0). Volumes of interest were drawn manually on the pancreas, tumours and the left cardiac ventricle (blood). The [^18^F]FDOPA uptake in tumours was expressed as time-activity curves of the pancreas and tumours normalised to blood radioactivity.

### Uptake and intratumoural distribution of [^18^F]FDOPA

The whole pancreata were exposed. Tumours were not dissected from the pancreata. Tumour volumes were calculated according to the formula *π*/6(*d*_1_ × *d*_2_ × *d*_3_), where *d*_1_ to *d*_3_ are perpendicular tumour diameters (mm) [30]. The absolute ^18^F radioactivity uptake was determined from blood and selected abdominal tissues (Table 1) using a gamma counter (3 in × 3 in NaI (Tl) crystal, Bicron 3 MW3/3P; Bicron Inc., Newbury, OH, USA) at 10 min after the injection of [^18^F]DOPA. Tissues were weighed, counted for radioactivity and corrected for background radioactivity and radioactivity decay. The quantity of radioactivity was expressed as the percentage of injected dose per gram of tissue (% ID/g). In order to determine the intratumoural biodistribution pattern of ^18^F radioactivity, tumours were rapidly frozen in dry ice/isopentane and cut with a cryomicrotome into 20-μm sections. Tissue sections were exposed to an imaging plate (Fuji BAS TR2025, Fuji Photo Film Co., Tokyo, Japan) for 4 ± 0.5 h. The spatial distribution of radioactivity was recorded with a phosphoimaging device (Fuji BAS 5000, with a resolution of 25 μm). Images were analysed for count density (photostimulated luminescence per unit area) with the Aida image analysis software (v. 4.22, Raytest Isotopenmessgeräte GmbH, Straubenhardt, Germany). The same tissue sections were stained with haematoxyline/eosin for histological analysis. Whole-tumour images were produced using a Zeiss AxioVert 200 M microscope with AxioCam MRc camera and the MosaiX option of AxioVision software (v. 4.8, Zeiss GmbH, Jena, Germany). Layers from histological images and autoradiography were overlaid using Adobe PhotoShop CS2 (San Jose, CA, USA), and the outlines of the tumours were drawn on autoradiography images according to the histological image.

### High-performance liquid chromatography

Samples of pancreata were taken near the tumour, homogenised into the high-performance liquid chromatography (HPLC) mobile phase/methanol and centrifuged, and the supernatant was used for metabolite analysis of [^18^F]FDOPA. The HPLC method consisted of a μBondapak C18 column (Waters Corporation, Milford, MA, USA) which was eluted with a solution of 50 mM sodium acetate, 20 mM citric acid, 1.0 mM sodium 1-octanesulfonate, 1.0 mM di-n-butylamine and 1.35 mM sodium EDTA in water/methanol (90:10 *v/v*). Authentic standards of [^18^F]3-OMFD, [^18^F]FDA, [^18^F]FMT, [^18^F]FDOPAC and [^18^F]FHVA [31,32] were used to identify retention times (Rt) in a similar chromatographic system.

### Statistical analysis

The mean weight of the sham-operated pancreas and pancreas with a tumour was compared with a two-sample *t* test. Radioactivity (ratios to non-target tissues and autoradiography) measurements were analysed using a two-way analysis of variance. Models included the main effects of pretreatment (vehicle, carbidopa and COMT + MAO-A) and group (sham-operated and tumour-bearing animals). In further pairwise comparisons between pretreatments, the Tukey adjustment method was used. Interaction between pretreatment and group was also tested. Due to positively skewed distributions, log-transformed values were used in statistical analyses. SAS System for Windows was used in statistical computations (v. 9.2, SAS Institute Inc., Cary, NC, USA); *p* values less than 0.05 were considered as statistically significant.

## Results and discussion

### Tumour characterisation and *ex vivo* biodistribution of [^18^F]FDOPA

No differences were detected between the weights of sham-operated and tumour-bearing mice (data not shown). No signs of cachexia were detected, which indicates that the tumours were not very large (data not shown). The tumour occurrence was 100%, and the mean tumour volume was 50 ± 40 mm^3^ at 35 days after tumour cell inoculation and 760 ± 1,300 mm^3^ at 42 days after tumour cell inoculation. The mean weight of the sham-operated pancreas was 0.18 ± 0.03 g, while the weight of the pancreas with a tumour at 35 and 42 days after inoculation was 0.27 ± 0.08 g (*p* < 0.001 vs. sham-operated) and 0.73 ± 0.97 g (*p* < 0.001 vs. sham-operated), respectively. As expected, the weight of the pancreata increased in tumour-bearing mice compared with the sham-operated mice.

The *ex vivo* distribution of [^18^F]FDOPA-derived activity was expressed as percentage of injected dose per gram of tissue (Table 1). The highest amount of radioactivity was found in the pancreas (including BxPC3 tumours, as applicable), liver, kidneys and small intestine when vehicle alone was used as pretreatment (Table 1). Interestingly, the uptake was twofold higher in sham-operated pancreata than in tumour-bearing pancreata (Table 1). Carbidopa pretreatment increased the uptake in the pancreas in sham-operated and tumour-bearing animals three- and fourfold, respectively, compared with vehicle pretreatment. However, no differences were detected between carbidopa pretreated sham-operated and tumour-bearing animals (Table 1). Combined administration of COMT and MAO-A inhibitors doubled the uptake of ^18^F radioactivity by the pancreata of sham-operated animals compared with vehicle pretreated animals. The ^18^F radioactivity uptake in the pancreas was threefold lower in tumour-bearing animals that received COMT + MAO-A-inhibitors in comparison with sham-operated animals with the same pretreatment. No major between-mice differences in other studied organs were detected. Administration of the MAO-A or COMT inhibitors alone had no effect on ^18^F radioactivity uptake (Table 1), and therefore, carbidopa and COMT + MAO-A inhibitors were selected for further study.

**Figure 2:**
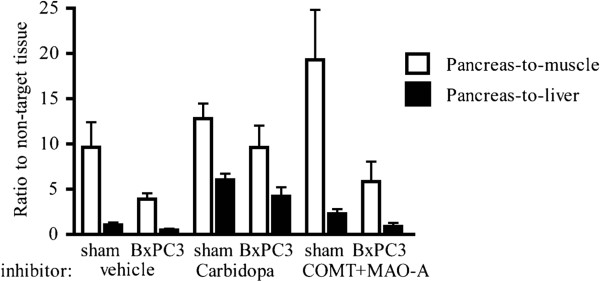
**Ratios to non-target tissues (pancreas-to-muscle and pancreas-to-liver) from *ex vivo *radioactivity measurements (% ID/g).** These were measured after [^18^F]FDOPA injection. Significant differences were detected between sham-operated and BxPC3 tumour-bearing pancreata (pretreatment adjusted *p* < 0.001 in pancreas-to-muscle and pretreatment adjusted *p* < 0.05 in pancreas-to-liver ratios), but pretreatment had no effect on these differences (pretreatment × group interaction effect, *p* = 0.245 in pancreas-to-muscle and *p* = 0.612 in pancreas-to-liver ratios). Carbidopa pretreatment increased uptake in the pancreata of both sham-operated and tumour-bearing mice compared with vehicle-treated mice (group adjusted *p* < 0.05 in pancreas-to-muscle and group adjusted *p* < 0.001 in pancreas-to-liver ratios). The numbers of mice exposed to vehicle, carbidopa and COMT + MAO-A inhibitors were 6, 5 and 4 for sham-operated animals, respectively, and 5, 7 and 4 for tumour-bearing animals, respectively. Values are shown as mean and standard error.

Target-to-non-target ratios (pancreas-to-muscle and pancreas-to-liver) were calculated based on measured radioactivities (% ID/g), and they are presented in Figure 2. Our data revealed significant differences between sham-operated and BxPC3 tumour-bearing pancreata (pretreatment adjusted *p* < 0.0001 in pancreas-to-muscle and *p* = 0.026 in pancreas-to-liver ratios). However, these differences were not dependent on the used pretreatment (pretreatment × group interaction effect, *p* = 0.245 in pancreas-to-muscle and *p* = 0.612 in pancreas-to-liver ratios). Carbidopa pretreatment increased the uptake in the pancreata in both sham-operated and tumour-bearing mice compared with vehicle treatment (group adjusted *p* = 0.037 in pancreas-to-muscle and *p* < 0.0001 in pancreas-to-liver ratios), but the difference between the sham-operated and tumour-bearing mice was not high enough to separate the healthy pancreas from the tumour-bearing pancreas (Figure 2). Pretreatment with COMT + MAO-A inhibitors increased the ratio further, especially the pancreas-to-muscle ratio, but due to the small number of observations and the larger variance in a measured radioactivity in the samples, the differences did not achieve any statistical significance (*p* = 0.133 in pancreas-to-muscle and *p* = 0.386 in pancreas-to-liver ratios, Figure 2).

### [^18^F]FDOPA PET imaging combined with [^18^F]FDG reveals xenograft tumours in mouse pancreas

**Figure 3:**
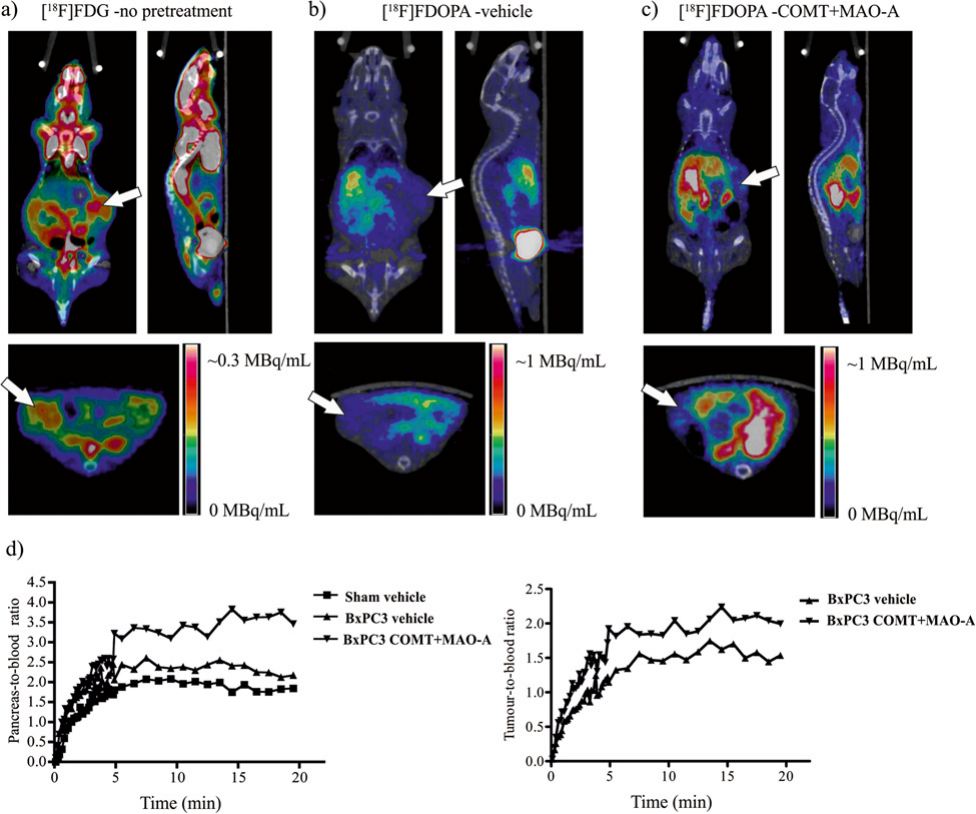
**Coronal, sagittal and transaxial PET/CT images of [**^**18**^**F]FDG (a) and [**^**18**^**F]FDOPA (b, c) and time-activity curves (d).** These are observed in mice bearing orthotopic pancreatic tumours (arrows); (**b**) and (**c**) images represent the sum of data collected 2 to 20 min after injection of [^18^F]FDOPA. Dark blue represents the lowest radioactivity, and red represents the highest radioactivity. Corresponding time-activity curves for (**b**) and (**c**) images are shown in (**d**), where pancreas and tumour signals are corrected with ratio to blood.

Mice were imaged with [^18^F]FDG PET/CT 6 weeks after tumour cell inoculation. Orthotopic pancreatic tumours exhibited increased glucose uptake (Figure 3a). As expected, high uptake of [^18^F]FDG was also observed in the heart, bladder, brain, kidneys and brown adipose tissue (Figure 3a). Next, the mice were imaged with [^18^F]FDOPA PET/CT. The uptake of [^18^F]FDOPA was very low in orthotopic BxPC3 tumours pretreated with vehicle (Figure 3b). However, the uptake in the healthy part of the pancreas increased after pretreatment with COMT + MAO-A inhibitors (Figure 3c,d). Based on 20 min of dynamic scanning, the peak and plateau radioactivities in the pancreas were reached within 5 min (Figure 3d). The time-activity curves of the pancreata showed similar dynamics regardless of pretreatment (vehicle vs. COMT + MAO-A inhibitors, Figure 3d) or tumour status (sham-operated vs. tumour-bearing mice, Figure 3d).

### Autoradiography identifies low [^18^F]FDOPA uptake in the tumour part of the pancreas

**Figure 4:**
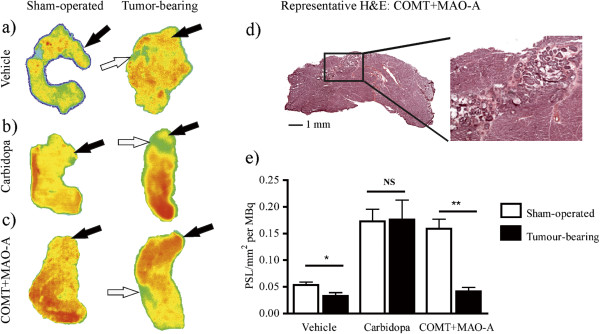
**Representative digital autoradiographs of pancreata, a corresponding haematoxylin and eosin-stained cryosection example and radioactivity measurements.** (**a**, **b**, **c**) In sham-operated and tumour-bearing pancreata 10 min after [^18^F]FDOPA injection, dark blue represents the lowest radioactivity, and red represents the highest radioactivity. Black arrows highlight the tail of the pancreas, and white arrows show the locations of the tumours. (**d**) An image of an example of the corresponding haematoxylin and eosin-stained cryosection was taken. (**e**) Radioactivity measurements were corrected for the dose of injected radioactivity. Pretreatment had effect on the difference between sham-operated and tumour-bearing animals (pretreatment × group interaction effect, *p* < 0.05). Significant differences were detected between sham-operated and tumour-bearing animals in vehicle (asterisk, *p* < 0.05) and COMT + MAO-A (double asterisks, *p* < 0.01) pretreated pancreata. Values are shown as mean and standard error.

Intratumoural distribution of ^18^F radioactivity was determined by digital autoradiography. Histological images of the haematoxylin/eosin-stained slices were combined with autoradiography, and the tumour outlines were drawn. In intrapancreatic comparison between the healthy pancreas and the tumour, radioactivity uptake was on average tenfold lower in tumour areas than in the healthy pancreas following pretreatments with vehicle or carbidopa. This uptake was fivefold lower in tumours of COMT + MAO-A pretreated pancreata (Figure 4a,b,c,d). In healthy areas of the pancreas, uptake was dependent on pretreatment. The uptake was uniform in the pancreata of vehicle- or carbidopa-treated mice (Figure 4a,b), while pretreatment with COMT + MAO-A inhibitors led to a 2.4-fold increase in uptake in the head of the pancreas compared with uptake in the tail of the pancreas (Figure 4c). A representative histological image indicates tumour growth in the body of the pancreas of mouse pretreated with COMT + MAO-A inhibitors (Figure 4d). After normalising the uptake with the amount of the injected radioactivity for each pancreas, pretreatment had an effect on the difference between sham-operated and tumour-bearing animals (pretreatment × group interaction effect *p* = 0.039, Figure 4e). Lower uptake was detected in tumour-bearing pancreata compared with sham-operated pancreata in vehicle (*p* = 0.016) and COMT + MAO-A inhibitor-treated (*p* = 0.002) animals. In carbidopa-treated mice, no significant difference was detected between sham-operated and tumour-bearing animals (*p* = 0.733). These observations were in accordance with *ex vivo* biodistribution data (Table 1 and Figure 2) as well as PET/CT data (Figure 3).

### Pretreatment affects [^18^F]FDOPA metabolism in pancreas

**Figure 5:**
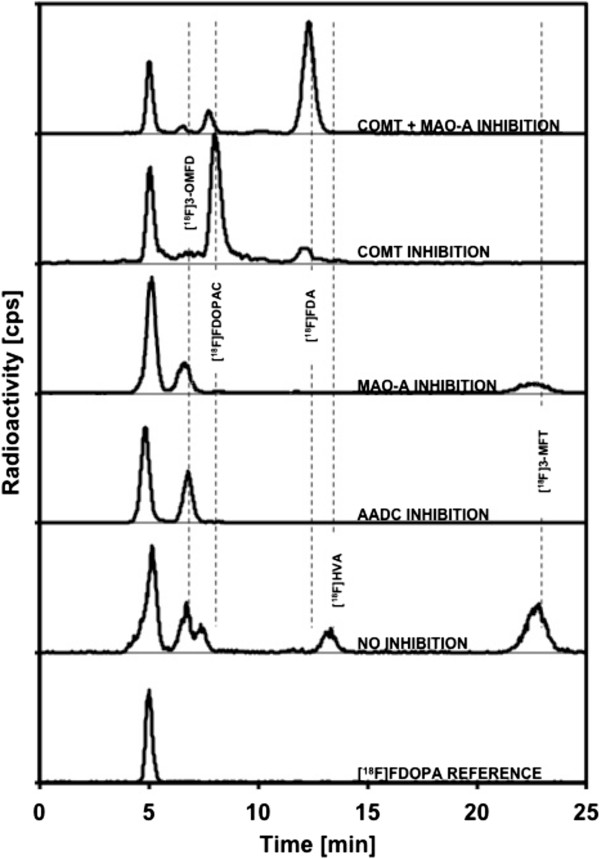
**Representative profiles of radioactive metabolites in pancreata after [**^**18**^**F]FDOPA injection.** Mice were treated with vehicle, carbidopa, COMT-inhibitor, MAO-A-inhibitor or COMT + MAO-A inhibitors.

Metabolites were analysed in pancreatic samples of sham-operated and tumour-bearing mice using HPLC. HPLC radiochromatograms from a radiometabolite study are shown in Figure 5. Of the several radiolabelled metabolites of [^18^F]FDOPA (Rt = 5 min), we identified [^18^F]3-OMFD (Rt = 7 min), [^18^F]FDOPAC (Rt = 8 min), [^18^F]FDA (Rt = 12 min), [^18^F]FHVA (Rt = 13 min) and [^18^F]FMT (Rt = 23 min). In the pancreata of vehicle-treated mice, three main metabolites were identified: [^18^F]3-OMFD, [^18^F]FHVA and [^18^F]FMT. Following the carbidopa pretreatment, the main metabolite in the samples was [^18^F]3-OMFD, while after MAO-A pretreatment, [^18^F]3-OMFD and [^18^F]FMT were identified. [^18^F]FDA was the major metabolite identified following pretreatment with COMT + MAO-A inhibitors. After the COMT pretreatment alone, the main metabolite was [^18^F]FDOPAC.

At present, the treatment of pancreatic adenocarcinoma is difficult because the location of the tumour lesion is usually unknown. In clinical practice, pancreatic cancer is imaged using [^18^F]FDG, which is the most widely used radiopharmaceutical for PET [33]. However, several factors may hamper imaging of the pancreas. Uptake of [^18^F]FDG in the duodenum may cause false-positive results, and imaging of the upper abdomen in general is influenced by respiratory and gastrointestinal tract motion [34]. Inflammatory cells are usually present in malignant lesions, further contributing to [^18^F]FDG uptake and leading to false-positive tumour-related findings [3]. [^18^F]FDOPA is a commonly used PET tracer for investigating the activity of the dopaminergic system in neurological disorders. [^18^F]FDOPA PET has also been successfully used to visualise neuroendocrine, carcinoid and glomus tumours as well as pheochromocytomas and medullary thyroid cancers [7,35-37]. The objective of this study was to investigate the usefulness of [^18^F]FDOPA also in the imaging of pancreatic adenocarcinoma and to assess the possible benefits of clinically available, widely used enzyme inhibitors of catecholamine neurotransmitters.

PET/CT was used to visualise whole-body anatomical and functional information from the tumour-bearing mice. Initially, orthotopic pancreatic cancer was imaged using [^18^F]FDG. [^18^F]FDG revealed increased glucose metabolism in the upper left abdomen of the tumour-bearing mice, and tumour lesions were determined based on the anatomical location (Figure 3a). Given the limitations of [^18^F]FDG, the same animals were imaged the next day using [^18^F]FDOPA in order to detect pancreatic tissue. As expected, [^18^F]FDOPA uptake occurred in the pancreata of these mice, but the uptake in pancreatic BxPC3 tumours was modest. According to our observations, combining [^18^F]FDG and [^18^F]FDOPA imaging improves the detection of cancerous tissue from the healthy pancreas.

Pretreatment with carbidopa or MAO and COMT inhibitors enhances the sensitivity of [^18^F]FDOPA brain imaging in Parkinson's disease patients as well as in abdominal tumour imaging [21,38]. In the present study, outlining of the pancreas improved, when the animals were treated with carbidopa or COMT and MAO-A inhibitors before [^18^F]FDOPA injection. All of these enzyme inhibitors prevent the breakdown of catecholamine neurotransmitters (Figure 1). Inhibition of AADC with carbidopa leads to the formation of [^18^F]3-OMFD (Figure 5), which is easily transported to both tumour cells and pancreatic cells [39]. However, when COMT + MAO-A were inhibited, the main metabolite is [^18^F]FDA (Figure 5), which is not taken up by BxPC3 tumour cells. Therefore, the uptake of ^18^F radioactivity is different between the healthy pancreas and tumour after pretreatment with different enzyme inhibitors.

*Ex vivo* distribution studies demonstrated major (two- to fourfold) differences in ^18^F-uptake ratios between sham-operated and tumour-bearing mice, which were pretreated with vehicle or COMT and MAO-A inhibitors prior to [^18^F]FDOPA injection. This observation can be explained by a change in [^18^F]FDOPA metabolism compared with that following carbidopa pretreatment. This was confirmed with tumour autoradiography. Although uptake was highest in carbidopa-treated pancreata, the difference between sham-operated and tumour-bearing pancreata was largest in animals pretreated with COMT + MAO-A inhibitors.

## Conclusions

Our study indicates that pretreatment of mice with carbidopa increases [^18^F]FDOPA uptake in the pancreas and therefore facilitates the localisation of the pancreas, but it also impairs the observer's ability to separate the healthy pancreas from the tumour because, in addition to exocrine pancreatic cells, tumour cells are also able to uptake [^18^F]3-OMFD, which is the main metabolite after carbidopa administration. COMT + MAO-A inhibitors increased the ^18^F radioactivity uptake in pancreatic tissue, while the uptake in tumours is poor due to the formation of [^18^F]FDA as main metabolite. Therefore, COMT + MAO-A inhibition improved the separation of the healthy pancreas from the tumour. However, the difference between the healthy pancreas and tumour was not clear in all cases after COMT + MAO-A administration, and the benefit over vehicle pretreatment remained modest. According to our observations, [^18^F]FDOPA combined with [^18^F]FDG imaging is a useful tool for detecting pancreatic adenocarcinoma, either alone or with COMT + MAO-A pretreatment, but not with carbidopa pretreatment. Since deducing the exact location of the tumour lesion is essential for a successful treatment, our data may have an important clinical value.

## Abbreviations

AADC: Aromatic amino acid decarboxylase; AD: Aldehyde dehydrogenase; Bq: Becquerel; COMT: Catechol-*O*-methyl transferase; F: Fluorine; [18F]FDA: 6-[^18^F]fluoro-l-3,4-dihydroxyphenylethylamine; [18F]FDG: 2-deoxy-2-[^18^F]fluoro-d-glucose; [18F]FDOPA: 6-[^18^F]fluoro-l-3,4-dihydroxyphenylalanine; [18F]FDOPAC: 6-[^18^F]fluoro-l-3,4-dihydroxyphenylacetic acid; [18F]FHVA: 6-[^18^F]fluorohomovanillic acid; [18F]3-OMFD: 6-[^18^F]fluoro-l-3-methoxy-4-hydroxyphenylalanine; ID: Injected dose; MAO: Monoamine oxidase; min: Minute; NaCl: Sodium chloride; PET/CT: Positron emission tomography/computed tomography; PSL: Photostimulated luminescence per unit area; Rt: Retention time

## Competing interests

The authors declare that they have no conflict of interest.

## Authors' contributions

MHS, JT and OS designed the study. JT made *in vivo* experiments, analysed autoradiography, gamma counter and PET/CT data and drafted the manuscript. LH did the HPLC quality control. TJG scanned the subjects. SF and OS prepared tracers. TV made statistical analyses. MHS did HPLC analyses and supervised all other analyses. All authors edited the manuscript. All authors read and approved the final manuscript.
